# A Case Report on Hiccups Improvement With Gabapentin

**DOI:** 10.7759/cureus.85849

**Published:** 2025-06-12

**Authors:** Ali Bahathiq, Marwah Abbas, Nojoud A Al Fareh

**Affiliations:** 1 Psychiatry, King Saud University, Riyadh, SAU; 2 Psychiatry, King Khalid University Hospital, Riyadh, SAU; 3 Psychiatry, King Khalid University Hospital, Abha, SAU

**Keywords:** case report, gaba, gabapentin, hiccups, persistent

## Abstract

Hiccups, or Singultus, are a common condition among individuals. Several medications are under research for hiccup management, including Gabapentin, an analog of the neurotransmitter γ-aminobutyric acid (GABA), which has shown effective outcomes in patients with persistent hiccups, highlighting the need for larger clinical studies to evaluate their efficacy and safety. Despite that, Gabapentin works on the α-2-δ subunit of calcium channels, mediating its effect. Like many hiccup treatments, the exact mechanism through which Gabapentin alleviates hiccups is not fully understood.

This case reports an 87-year-old male with a 40-year history of chronic hiccups, unresponsive to traditional psychiatric treatments, including chlorpromazine and haloperidol. The patient began escitalopram 20 mg daily for low mood and later added gabapentin 300 mg three times a day, which significantly improved his hiccups to under 15 minutes daily without any side effects. This case highlights the efficacy and safety of Gabapentin as an effective and well-tolerated treatment for persistent hiccups of unknown cause.

## Introduction

Hiccups, or Singultus, are a common condition among individuals. However, it is still not a fully understood phenomenon. It occurs when there is a sudden, involuntary contraction of the diaphragm and chest muscles, followed by the closure of the larynx. This causes air to rush into the lungs and the vocal cords to close, resulting in the "hic" sound, which is heard between four and 60 times per minute [1,2]. Hiccups are caused by a 'reflex arc' with afferent, central, and efferent components. They are mostly triggered by stomach distension due to large meals or carbonated drinks, spicy foods, alcohol, smoking, and other irritants. Anxiety or stress accompanied by over-breathing can also trigger hiccups. In addition, neurological and cardiovascular disease, alterations to the electrolyte balance, and medications such as midazolam (benzodiazepines), morphine (opiates), and dexamethasone (steroids) can be associated with persistent hiccups [2,3]. Hiccups are classified according to the duration of episodes [4]. Acute attacks last 48 hours or less. "Persistent hiccups" last over two days. "Intractable hiccups" occur when an attack persists for over a month [5].

Hiccups may not be a serious condition, but effective management relies on addressing the underlying cause. Various medications have been studied to manage persistent and uncontrollable hiccups. Nevertheless, insufficient high-quality research suggests specific hiccup therapies [6,7]. The hiccup reflex is processed centrally by γ-aminobutyric acid (GABA), dopamine, and serotonin [8,9]. Dopaminergic and GABAergic receptors are the targets of most pharmaceutical therapies. Chlorpromazine remains the only Food and Drug Administration (FDA) approved medication for hiccups in the USA, yet it may not be suitable for all patients, particularly older adults, due to its severe side effects, including irreversible movement disorders. Additionally, only two drugs have been studied in small, randomized controlled trials (RCTs), including metoclopramide and baclofen [10,11]. Other medications, such as amitriptyline, haloperidol, midazolam, nifedipine, nicodipine, and valproic acid, have been explored in small studies but are not yet incorporated into clinical guidelines [12].

Gabapentin, an analog of the inhibitory neurotransmitter GABA, has emerged as a promising alternative. It has shown effectiveness for outcomes for persistent hiccup patients. Studies on Gabapentin’s efficacy are limited to case reports, and its exact mechanism of action in hiccup resolution remains unclear [13].

This case report aims to present a rare case of a 87-year-old male with a 40-year history of chronic intractable hiccups successfully managed with Gabapentin, underscoring the potential of this medication as a therapeutic option when conventional treatments fail.

## Case presentation

This is an 87-year-old man who is married to two wives, each with ten daughters and six sons. He lives in Al-Kharj, is uneducated, and is unemployed. The patient has been experiencing hiccups since 1970. The hiccups episodes would last for several days and then remit for months. The episodes showed improvement with antacid medications, while there was no apparent association with food intake or psychological stress. Since 2015, he has suddenly started experiencing daily hiccups for more than 10 hours, unrelated to eating, even while fasting, and with no clear trigger factors. The patient had no use of illicit substances, alcohol, or smoking.

The patient has hypertension, dyslipidemia, paroxysmal atrial fibrillation on rivaroxaban, ischemic heart disease (IHD), and underwent percutaneous coronary intervention with a stent in 2018. He had a hip fracture in 2020 and underwent robotic hiatal hernia repair and Toupet fundoplication, but it was unsuccessful, prompting a referral to a psychiatrist.

Regarding his past psychiatric history, he has no prior psychiatric diagnosis or treatment with psychotropic medications; no current or past history of social phobia or other phobias; no past or present repetitive thoughts or actions; no passive or active suicidal or self-harming thoughts or plans; no current or past history of eating disorders or attention deficit hyperactivity disorder (ADHD). Only bouts of low mood are associated with hiccups. The patient is self-reliant in daily tasks, used to working in sheep farming, and drinks camel milk boiled with honey. There is no family history of psychiatric disorders.

The patient tried various psychiatric medications: first, chlorpromazine 25 mg orally (PO) three times a day (TID) for two months before being admitted to our hospital, with no improvement. Then, from 26 October 2020 to 21 January 2021, he started haloperidol 0.5 mg for another two months, also with no improvement. On 4 March 2021, the patient was maintained on escitalopram 20 mg PO once daily (OD) for low mood. On 3 June 2021, the patient was prescribed Gabapentin 300 mg PO TID, which reduced the symptoms of hiccups to less than a quarter of an hour per day without any side effects. The patient’s full management plan and its outcome for his chronic hiccups are detailed in Table 1.

**Table 1:** Patient’s management plan and outcome for hiccups BID: two times a day;  OD: once daily; PO: per oral; TID: three times a day.

Date	Ordered	Maintained Dose	Outcome
From 26-10-2020 till 21-01-2021	Discontinue chlorpromazine and start haloperidol	0.5 mg PO BID	- No improvement
04-03-2021	Escitalopram	20 mg PO OD	- Mood improvement
03-06-2021	Gabapentin	300 mg PO TID	- Hiccups symptoms reduced without any side effects - Same improvement till 27-6-2024 on the same medications

The patient's last follow-up was on 27 June 2024, at which point they were clinically stable, and the gabapentin dose was tapered to 300 mg PO TID. Selected laboratory values during gabapentin therapy from August 2022 (Table 2), with some abnormalities, such as prolonged PT/INR and hypoalbuminemia, were present; their clinical significance in relation to gabapentin use or the patient’s hiccups could not be definitively determined.

**Table 2:** Patient selected lab values following gabapentin treatment (August 2022) MCV: mean corpuscular volume; PT: prothrombin time; INR: international normalized ratio; APTT: activated partial thromboplastin time; ALK Phos: alkaline phosphatase.

Parameter	24/8/2022	22/8/2022	21/8/2022	16/8/2022	Normal range
MCV	94.1 (fL)	94.8 (fL)	99.3 (fL)	95.8 – 95.9 (fL)	80–98 (fL)
PT	20.20 (seconds)	15.20 (seconds)	15.10 (seconds)	35.00-36.80 (seconds)	11–13 (seconds)
INR	1.48	1.11	1.09	2.68 – 2.82	0.9-1.2
APTT	50.90 (seconds)	38.80 (seconds)	38.80 (seconds)	48.80 – 61.10 (seconds)	25–35 (seconds)
Albumin	25.90 (g/L)	28.50 (g/L)	27.10 (g/L)	29.50 – 28.80 (g/L)	35–55 (g/L)
Calcium	2.02 (mmol/L)	1.92 (mmol/L)	1.90 (mmol/L)	1.94 (mmol/L)	2-2.6 (mmol/L)
Sodium	128.41 (mEq/L)	133.1 (mEq/L)	133.59 (mEq/L)	129-130.67 (mEq/L)	136–145 (mEq/L)
ALK Phos	173 (U/L)	138 (U/L)	137 (U/L)	92 – 106 (U/L)	30–120 (U/L)

## Discussion

Gabapentin has been reported to be more effective for hiccups of central nervous system (CNS) origin than for those arising from gastrointestinal (GI) causes [6]. However, in this patient, while the positive response to gabapentin may suggest CNS involvement, the definitive origin of hiccups, CNS, GI, or mixed, remains uncertain, particularly given the history of hiatal hernia repair.

In this case, the patient suffered from hiccups for 40 years, indicating the severity of the physical and social burden caused to the patient over this long duration. The treatment plan of Gabapentin prescribed for this patient was effective. Complete improvement was achieved with Gabapentin rather than haloperidol. By the patient’s last follow-up on 27 June 2024, the patient remained stable, and the dosage was reduced to 300 mg PO TID, with no recurrence of symptoms observed during the evaluation period. This case is noteworthy as it illustrates the potential efficacy of gabapentin in managing persistent hiccup symptoms, supporting its consideration as a therapeutic option in similar clinical scenarios.

Several medications are under research for hiccup management, including baclofen, gabapentin, and gastroprokinetic metoclopramide, which all require the development of larger clinical studies to evaluate their efficacy and safety among larger and representative populations. Neither Guidelines nor large RCTs have been conducted on the clinical management of chronic hiccup patients [14]. 

Gabapentin is an analog of the neurotransmitter GABA and works on the α-2-δ subunit of calcium channels, mediating its effect [15]. Therefore, Gabapentin has been effective in many neurological diseases, such as fibromyalgia, epilepsy, neuropathic pain, and postoperative pain [16]. The exact mechanism through which Gabapentin improves hiccups is not fully understood in the literature [6].

Previous research has identified many case reports of patients using Gabapentin to manage hiccups. In 2023, a hiccup case that lasted over four days in a patient with acute myeloblastic leukemia was successfully treated with Gabapentin [17]. In 2017, another case report of a 54-year-old man who underwent pulsed radiofrequency treatment and was suffering from persistent hiccups was finally managed with Gabapentin 300 mg twice daily. The outcome of this case showed the complete resolution of symptoms within 30 days, suggesting that Gabapentin may be effective in such cases [18]. A systematic search was developed in 2013 to study the efficacy of Gabapentin in hiccups. A few clinical trials, including 17 case reports and two case series incorporating gabapentin therapy for persistent or intractable hiccups, were assessed [14]. Nine cases could be defined as intractable, while three were persistent. Gabapentin doses varied from 200 to 1200 mg per day, and treatment durations ranged from a single day to ongoing use.

Combination therapy and comorbidities were presented in some cases. Previous case reports suggested that Gabapentin might be used as a second-line medication in patients undergoing stroke rehabilitation or those in the palliative care setting, where using chlorpromazine is not possible due to undesirable adverse effects. Such cases are very rare; the last case was discovered in 2012. In addition, the duration of hiccups among patients varied among the included cases, with 50 years reported as the longest duration in 2007 [19]. 

Initial treatments in this case included chlorpromazine, haloperidol, and escitalopram; however, these medications failed to achieve symptom relief. Chlorpromazine and haloperidol, while traditionally used for hiccup management, may not have been effective due to the refractory nature of the condition or other patient-specific factors. Although escitalopram has been rarely associated with hiccups, its confounding role appears unlikely as symptoms improved with gabapentin titration while the escitalopram dose remained unchanged. However, potential CNS-mediated interactions between these medications cannot be completely excluded. Gabapentin's success can be attributed to its modulation of neuronal excitability, though its exact mechanism in resolving hiccups remains unclear. This highlights its potential as a viable treatment option for refractory hiccups, warranting further investigation.

Overall, the evidence shows positive clinical outcomes with Gabapentin in all included studies. Gabapentin was very well tolerated, with only a few minor adverse effects. Some of its adverse effects include dizziness, fatigue, ataxia, and peripheral edema. Neurologic toxicity, painful gynecomastia, or hypoglycemia are among the rare adverse effects that can be associated with the use of Gabapentin [20]. Finally, Gabapentin's oral absorption is mediated by both facilitated and passive absorption. The highest absolute bioavailability is achieved at 900 mg, reaching 60%. As the dosage increases from 900 to 3600 mg/day, the absolute bioavailability declines from 60% to 33% [21].

In this case, laboratory findings during gabapentin therapy-including hypoalbuminemia (25.9-29.5 g/L), hypocalcemia (1.90-2.02 mmol/L), mild hyponatremia (128-133 mEq/L), and elevated prothrombin time (PT)/ international normalized ratio (INR)/ activated partial thromboplastin time (APTT), highlight the need for comprehensive metabolic and coagulation monitoring in medically complex patients treated for refractory hiccups. Future reports would benefit from closer laboratory monitoring and standardized follow-up to better evaluate the safety profile and therapeutic response to gabapentin in patients with persistent hiccups, especially in those with multiple comorbidities.

## Conclusions

This case report supports earlier studies demonstrating the effectiveness of Gabapentin in treating persistent hiccups in elderly patients. Gabapentin is safe, well-tolerated, available, and free from severe adverse effects, offering a therapeutic advantage over antidopaminergic drugs, which may cause severe and irreversible side effects in adults. Its low pharmacological interaction further underscores its suitability for elderly patients. Therefore, further research on larger populations is needed to refine the management plan for persistent hiccups, aiming to enhance the quality of life for patients with this condition.

## References

[1] Kahrilas PJ, Shi G (1997). Why do we hiccup?. Gut.

[2] Chang FY, Lu CL (2012). Hiccup: mystery, nature and treatment. J Neurogastroenterol Motil.

[3] Rey E, Elola-Olaso CM, Rodríguez-Artalejo F, Locke GR 3rd, Díaz-Rubio M (2006). Prevalence of atypical symptoms and their association with typical symptoms of gastroesophageal reflux in Spain. Eur J Gastroenterol Hepatol.

[4] Kolodzik PW, Filers MA (1991). Hiccups (singultus): review and approach to management. Ann Emerg Med.

[5] Cymet TC (2002). Retrospective analysis of hiccups in patients at a community hospital from 1995-2000. J Natl Med Assoc.

[6] Steger M, Schneemann M, Fox M (2015). Systemic review: the pathogenesis and pharmacological treatment of hiccups. Aliment Pharmacol Ther.

[7] Hashiguchi M, Fujita A, Ikeda M, Morikawa M, Kohmura E (2018). Intractable hiccups after coil embolization of partially thrombosed posterior inferior cerebellar artery aneurysm. World Neurosurg.

[8] Cole JA, Plewa MC (2024). Singultus. StatPearls [Internet].

[9] Sampath V, Gowda MR, Vinay HR, Preethi S (2014). Persistent hiccups (singultus) as the presenting symptom of lateral medullary syndrome. Indian J Psychol Med.

[10] Zhang C, Zhang R, Zhang S, Xu M, Zhang S (2014). Baclofen for stroke patients with persistent hiccups: a randomized, double-blind, placebo-controlled trial. Trials.

[11] Wang T, Wang D (2014). Metoclopramide for patients with intractable hiccups: a multicentre, randomised, controlled pilot study. Intern Med J.

[12] Choi TY, Lee MS, Ernst E (2012). Acupuncture for cancer patients suffering from hiccups: a systematic review and meta-analysis. Complement Ther Med.

[13] Thompson DF, Brooks KG (2013). Gabapentin therapy of hiccups. Ann Pharmacother.

[14] Kohse EK, Hollmann MW, Bardenheuer HJ, Kessler J (2017). Chronic hiccups: an underestimated problem. Anesth Analg.

[15] Gajcy K, Lochyński S, Librowski T (2010). A role of GABA analogues in the treatment of neurological diseases. Curr Med Chem.

[16] Calandre EP, Rico-Villademoros F, Slim M (2016). Alpha(2)delta ligands, gabapentin, pregabalin and mirogabalin: a review of their clinical pharmacology and therapeutic use. Expert Rev Neurother.

[17] Alshammary SA, Al Fraihat LA, Farahat YH, Alshehri A, Almustanyir S (2023). Successful treatment of persistent hiccups in an advanced palliative cancer patient with gabapentin: a case report. Cureus.

[18] Ozturk O, Yavuz E, Yazicioglu B, Uzuner B (2017). Treatment of resistant idiopathic hiccups with pulse radio frequency on phrenic nerve and gabapentin: a case report. Niger J Clin Pract.

[19] Carlisi E, Bossi D, Zaliani A, Dalla Toffola E (2012). Persistent hiccup after surgical resection of a brainstem arteriovenous malformation: a case successfully treated with gabapentin during rehabilitation. Case report. Eur J Phys Rehabil Med.

[20] Quintero GC (2017). Review about gabapentin misuse, interactions, contraindications and side effects. J Exp Pharmacol.

[21] Bockbrader HN, Wesche D, Miller R, Chapel S, Janiczek N, Burger P (2010). A comparison of the pharmacokinetics and pharmacodynamics of pregabalin and gabapentin. Clin Pharmacokinet.

